# Association of Erythropoietin-Stimulating Agent Responsiveness with Mortality in Hemodialysis and Peritoneal Dialysis Patients

**DOI:** 10.1371/journal.pone.0143348

**Published:** 2015-11-20

**Authors:** Myoung Nam Bae, Su Hyun Kim, Young Ok Kim, Dong Chan Jin, Ho Chul Song, Euy Jin Choi, Yong-Lim Kim, Yon-Su Kim, Shin-Wook Kang, Nam-Ho Kim, Chul Woo Yang, Yong Kyun Kim

**Affiliations:** 1 Department of Internal Medicine, College of Medicine, The Catholic University of Korea, Seoul, Korea; 2 Department of Internal Medicine, College of Medicine, Chung-Ang University, Seoul, Korea; 3 Department of Internal Medicine, School of Medicine, Kyungpook National University, Daegu, Korea; 4 Department of Internal Medicine, College of Medicine, Seoul National University, Seoul, Korea; 5 Department of Internal Medicine, College of Medicine, Yonsei University, Seoul, Korea; 6 Department of Internal Medicine, Chonnam National University Medical School, Kwangju, Korea; 7 MRC for Cell Death Disease Research Center, The Catholic University of Korea, Seoul, Korea; Robert Bosch Hospital, GERMANY

## Abstract

Erythropoiesis-stimulating agent (ESA) responsiveness has been reported to be associated with increased mortality in hemodialysis (HD) patients. ESA requirement to obtain the same hemoglobin (Hb) level is different between HD and peritoneal dialysis (PD) patients. In this study, we investigated the impact of ESA responsiveness on mortality between both HD and PD patients. Prevalent HD and PD patients were selected from the Clinical Research Center registry for end-stage renal disease, a prospective cohort study in Korea. ESA responsiveness was estimated using an erythropoietin resistant index (ERI) (U/kg/week/g/dL). Patients were divided into three groups by tertiles of ERI. ESA responsiveness was also assessed based on a combination of ESA dosage and hemoglobin (Hb) levels. The primary outcome was all-cause mortality. A total of 1,594 HD and 876 PD patients were included. The median ESA dose and ERI were lower in PD patients compared with HD patients (ESA dose: 4000 U/week vs 6000 U/week, respectively. P<0.001, ERI: 7.0 vs 10.4 U/kg/week/g/dl, respectively. P<0.001). The median follow-up period was 40 months. In HD patients, the highest ERI tertile was significantly associated with higher risk for all-cause mortality (HR 1.96, 95% CI, 1.07 to 3.59, P = 0.029). HD patients with high-dose ESA and low Hb levels (ESA hypo-responsiveness) had a significantly higher risk of all-cause mortality (HR 2.24, 95% CI, 1.16 to 4.31, P = 0.016). In PD patients, there was no significant difference in all-cause mortality among the ERI groups (P = 0.247, log-rank test). ESA hypo-responsiveness was not associated with all-cause mortality (HR = 1.75, 95% CI, 0.58 to 5.28, P = 0.319). Our data showed that ESA hypo-responsiveness was associated with an increased risk of all-cause mortality in HD patients. However, in PD patients, ESA hypo-responsiveness was not related to all-cause mortality. These finding suggest the different prognostic value of ESA responsiveness between HD and PD patients.

## Introduction

Correction of severe anemia toward the conventional hemoglobin (Hb) target level using erythropoiesis-stimulating agents (ESA) has beneficial effects on the reduction of left ventricular mass in patients with hemodialysis (HD) [1–5]. In spite of the beneficial effect of ESA on cardiovascular prognosis, the survival benefit provided by the ESA-induced increase in Hb levels has been questioned [6,7]. Previous studies have reported that the response to ESA treatment is associated with the survival rate, and that hypo-responsiveness to ESA treatment is a known predictor of poorer outcome in patients on HD [8–12].

The prevalence and severity of anemia is lower in patients on peritoneal dialysis (PD) than in patients on HD [13,14]. Furthermore, patients on PD have lower requirements of ESA to obtain the same Hb level, compared with patients on HD [15–17]. For these reasons, it may be postulated that the impact of the response to ESA treatment on mortality may differ between patients with HD and PD. For the association of ESA hypo-responsiveness and mortality in PD patients, US study and the Netherland Cooperative study on the Adequacy of Dialysis (NECOSAD) study reported that ESA hypo-responsiveness was associated with higher morality in both PD patients and HD patients [16,17]. Interestingly, US study showed the different pattern of ESA dose on mortality between patients with HD and PD [17]. In PD patients, there was some increased risk only when the ESA doses exceeded 15,000 U/week, while the association of ESA dose with mortality was linear, robust and incremental in HD patients [17]. Furthermore, considering of international differences of trends in ESA use and Hb levels in dialysis patients [18,19], the impact of ESA responsiveness on mortality in HD and PD patients may vary among the countries and ethnic group.

In this study, we investigated the impact of ESA responsiveness on all-cause mortality in the HD and PD populations in the Clinical Research Center (CRC) registry for end-stage renal disease (ESRD) cohort, an observational prospective cohort study conducted in Korea.

## Materials and Methods

### Study Population

All patients in this study participated in the CRC for ESRD. This is an ongoing observational prospective cohort study in patients with ESRD from 31 centers in Korea. The cohort was established in April 2009 and includes adult (>18 years of age) dialysis patients. A total of 1,811 prevalent patients on HD and 1,174 prevalent patients on PD were enrolled in this cohort. For the present study, we excluded patients for whom information about their ERI was not available (n = 217, n = 298). Finally, 1,594 HD and 876 PD patients were included in the final analysis. The CRC registry for ESRD was approved by the medical ethics committees of all participating hospitals and informed consent was obtained from all patients before inclusion.

### Ethics

This study was approved by the institutional review boards at each center. The names of the institutional review boards were as follow. The Catholic University of Korea, Bucheon St. Mary's Hospital; The Catholic University of Korea, Incheon St. Mary's Hospital; The Catholic University of Korea, Seoul St. Mary's Hospital; The Catholic University of Korea, St. Mary's Hospital; The Catholic University of Korea, St. Vincent's Hospital; The Catholic University of Korea, Uijeongbu St. Mary's Hospital; Cheju Halla General Hospital; Chonbuk National University Hospital; Chonnam National University Hospital; Chung-Ang University Medical Center; Chungbuk National University Hospital; Chungnam National University Hospital; Dong-A University Medical Center; Ehwa Womens University Medical Center; Fatima Hospital, Daegu; Gachon University Gil Medical Center; Inje University Pusan Paik Hospital; Kyungpook National University Hospital; Kwandong University College of Medicine, Myongji Hospital; National Health Insurance Corporation Ilsan Hospital; National Medical Center; Pusan National University Hospital; Samsung Medical Center, Seoul; Seoul Metropolitan Government, Seoul National University, Boramae Medical Center; Seoul National University Hospital; Seoul National University, Bundang Hospital; Yeungnam University Medical Center; Yonsei University, Severance Hospital; Yonsei University, Gangnam Severance Hospital; Ulsan University Hospital; Wonju Christian Hospital (in alphabetical order). This study was performed in accordance to the 2008 Declaration of Helsinki. Written informed consent was obtained from all patients before inclusion.

### Data Collection

Baseline demographic and clinical data including age, gender, height, weight, systolic blood pressure (BP), diastolic BP, co-morbidities, laboratory investigations, nutritional status and therapeutic characteristics were recorded. Serum Hb, total cholesterol (TC), albumin, calcium, phosphorus and intact parathyroid hormone (iPTH) levels were determined from blood samples. The single-pool Kt/V (spKt/V) was determined by two-point urea modeling based on the intradialytic reduction in blood urea and intradialytic weight loss [20]. The calculation of weekly Kt/V was performed by standard methods, using data from 24-hour dialysate and urine collections [21]. For the assessment of co-morbidity, a modified Charlson comorbidity score was used [22]. Nutritional status measured by subject global assessment of nutritional status (SGA): the well-nourished group had a score of 6–7, the mild-moderate malnourished group a score of 3–5, and the severe malnourished group a score of 1–2. The darbepoietin doses were harmonized with erythropoietin data by multiplying by 200 [23].

For the assessment ESA responsiveness, we used the ESA resistance index (ERI), calculated as the weekly weight-adjusted dose of ESA (U/kg/week) divided by the Hb concentration (g/dL) [5,8]. HD and PD patients were divided into 3 groups, by tertiles of ERI, as follows: HD patients; Tertile 1, ERI < 6.57; Tertile 2, 6.57 ≤ ERI < 14.74; and Tertile 3, ERI ≥ 14.74 and PD patients; Tertile 1, ERI < 4.06; Tertile 2, 4.06 ≤ ERI < 10.1; and Tertile 3, ERI ≥ 10.1 U/kg/week/g/dL.

We also categorized ESA responsiveness based on a combination of ESA dosage (high: ≥ median value of ESA dose or low: < median value of ESA dose) and Hb levels (high: ≥10 g/dL or low: <10 g/dL). Thus, ESA responsiveness was divided to 4 categories combining the categorical determinants of ESA dosage (2 groups) and Hb levels (2 groups).

### Outcomes

The primary outcome of this study was all-cause mortality. For each death, the principal investigator at the given institution completed a form that included cause of death according to the CRC registry for ESRD study classification. Dates and causes of mortality were immediately reported throughout the follow-up period.

### Statistical Analyses

Data with continuous variables and a normal distribution are presented as mean ± SD and those without a normal distribution are presented as the median with ranges as appropriate for the type of variable. Student’s t-tests, Mann-Whitney tests, One-way ANOVA tests or Kruskal-Wallis tests were used, as appropriate, to determine the differences in continuous variables. Categorical variables are presented as percentages. Pearson’s chi-square tests or Fisher’s exact tests were used to determine the differences in categorical variables.

Absolute mortality rates were calculated per 100 person-years of follow-up. Survival curves were estimated by the Kaplan-Meier method and compared by the log-rank test according to the ERI categories. The Cox proportional hazards regression model was used to calculate a hazard ratio (HR) with a 95% confidence interval (CI) for all-cause mortality, using the ERI of tertile 1 as the reference value. A value of p<0.05 was considered statistically significant. Statistical analyses were performed using SPSS 18 software (Chicago, IL, USA).

## Results

### Patient Characteristics

A total of 1,594 HD and 876 PD patients were included. The baseline characteristics of the HD and PD patients enrolled as subjects in this study are shown in Table 1. The mean ages of HD and PD patients were 58±13 years and 54±12 years, respectively. The prevalence of diabetes and previous cardiovascular disease was lower in PD patients. The modified Charlson co-morbidity score was lower in PD patients compared with HD patients. Hb levels showed no significant difference between HD and PD group. The ESA dose was lower in PD patients compared with HD patients (4000 U/week vs 6000 U/week, respectively. *P*<0.001). The ERI was lower in PD patients than in HD patients (7.0 U/kg/week/g/dl vs 10.4 U/kg/week/g/dl, respectively. *P*<0.001). The most common ESAs used are Epoetin alfa and Darbepoietin alfa. The use of Epoetin alfa in HD patients is higher than PD patients. There was no significant difference in the use of Epoetin beta and Darbepoietin alfa between HD and PD patients.

**Table 1:** Clinical Characteristics of the Study Population According to Dialysis Modality.

	HD (n = 1594)	PD (n = 876)	P value
**Male, n (%)**	904 (56.7)	498 (56.8)	0.491
**Age, years**	58 ± 13	54 ± 12	<0.001
**DM, n (%)**	773 (48.5)	326 (37.2)	<0.001
**Previous CVD history, n (%)**	286 (17.9)	122 (13.9)	0.002
**Modified CCI**	4.97 ± 2.13	4.21 ± 1.89	<0.001
**SGA, n (%)**			0.079
** Well-nourished**	1261 (79.1)	625 (71.3)	
** Mild-moderate**	225 (14.1)	98 (11.2)	
** Severe malnourished**	0 (0)	2 (0.2)	
**Iron treatment, n (%)**			<0.001
** Oral iron treatment**	934 (58.6)	561 (64.0)	
** IV iron treatment**	187 (11.7)	14 (1.6)	
** No iron treatment**	465 (29.2)	279 (31.8)	
**Duration of dialysis, months**	33.8 (14.0–65.9)	31.2 (14.4–62.3)	0.067
**Systolic BP, mmHg**	141.0 ± 20.7	132.4 ± 21.3	<0.001
**Diastolic BP, mmHg**	76.8 ± 12.7	79.5 ± 12.4	<0.001
**Body weight, kg**	59.3 ± 10.6	62.8 ± 11.0	<0.001
ESA dose, *10 ^**^3**^ **U/week**	6 (4–10)	4 (0–8)	<0.001
**ERI, U/kg/week/g/dl**	10.4 (5.2–17.5)	7.0 (0–12.0)	<0.001
**Hemoglobin, g/dl**	10.7 ± 1.2	10.6 ± 1.5	0.655
**Iron, mcg/dl**	68.6 ± 33.3	72.9 ± 33.6	0.008
**TSAT, %**	31.5 ± 15.9	31.3 ± 15.2	0.762
**Ferritin, ng/ml**	292.1 ± 310.4	287.2 ± 323.3	0.748
**Albumin, g/dl**	3.90 ± 0.39	3.65 ± 0.47	<0.001
**Calcium, mg/dl**	8.81 ± 0.87	8.67 ± 0.84	<0.001
**Phosphorus, mg/dl**	5.45 ± 16.0	4.98 ± 1.51	0.384
**Intact PTH, pg/ml**	197.5 ± 231.1	309.6 ± 327.1	<0.001
**hsCRP, mg/dl**	1.66 ± 5.28	0.59 ± 2.38	<0.001
**Total cholesterol, mg/dl**	152.8 ± 36.1	175.5 ± 40.2	<0.001
**Ccr, ml/min**	1.23 ± 4.34	2.27 ± 5.34	<0.001
**Single-pool Kt/V**	1.48 ± 0.42		
**Weekly Kt/V**		1.64 ± 0.54	

Data are expressed as means SD, medians (interquartile range) or numbers (percentages), as appropriate.

BP, blood pressure; CVD, cardiovascular diseases; CCI, Charlson co-morbidity index; Ccr, creatinine clearance; ESA, erythropoiesis-stimulating agent; HD, hemodialysis; hsCRP, high sensitivity C-reactive protein; IV, intravenous; Kt/V: K, dialyzer clearance; t, time; V, volume of water a patient’s body contains; PD, peritoneal dialysis; PTH, parathyroid hormone; SGA, subject global assessment; TSAT, transferrin saturation.

With respect to iron treatment, oral iron therapy use was more prevalent in PD patients than in HD patients, while intravenous iron therapy showed a lower rate in PD patients than in HD patients. Serum iron levels were higher in PD patients and there were no significant differences in transferrin saturation (TSAT) and serum ferritin in either group. Serum albumin and high sensitivity C-reactive protein (hsCRP) levels were higher in HD patients.

**Fig 1:**
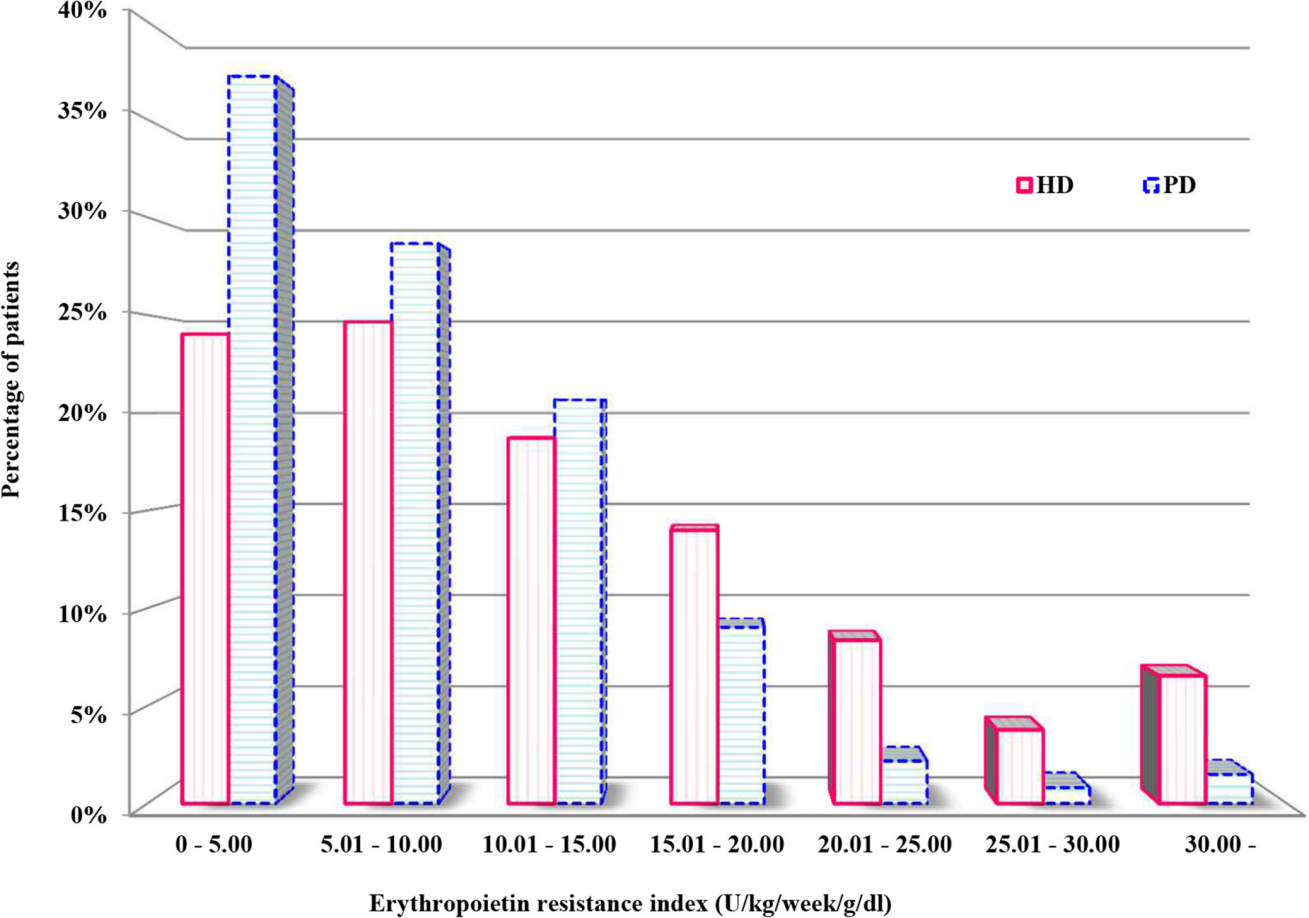
Distribution of patients according to erythropoietin resistance index in hemodialysis and peritoneal dialysis patients.

Fig 1 shows the distribution of patients according to their ERI in HD and PD patients. At ERI values of less than 15 U/kg/week/g/dL, PD patients were more prevalent than HD patients, while HD patients were more prevalent that ERI values above 15 U/kg/week/g/dL.

Table 2 shows the baseline characteristics of patients by ERI tertiles according to dialysis modality. Among HD patients, patients in the highest ERI tertile were more likely to be female, receive intravenous iron therapy, undergo a longer period of dialysis therapy, have higher serum ferritin levels, hsCRP, spKt/V and have lower prevalence of previous CVD history, SGA scores, body weight, and serum levels of hemoglobin, iron and albumin. There were no significant differences in age, prevalence of diabetes, modified Charlson co-morbidity score, or serum levels of TAST, iPTH and TC. Among PD patients, patients within the highest ERI tertile were more likely to be female, younger than lower ERI tertile groups, had lower body weights and lower serum levels of hemoglobin, iron and albumin. Patients within the higher ERI tertile had a higher iPTH and weekly Kt/V. There were no significant differences in prevalence of diabetes and cardiovascular diseases, modified Charlson co-morbidity score, SGA score, iron treatment, or serum levels of ferritin, hsCRP, and TC.

**Table 2:** Clinical characteristics of the study population according to tertiles of ERI.

	HD				PD			
	ERI (U/kg/week/g/dl)				ERI (U/kg/week/g/dl)			
	Tertile 1 (ERI<6.57)	Tertile 2 (6.57≤ERI<14.74)	Tertile 3 (ERI≥14.74)	P	Tertile 1 (ERI<4.06)	Tertile 2 (4.06≤ERI<10.1)	Tertile 3 (ERI≥10.1)	P
**Patient number**	531	532	531		291	292	293	
**Male, n (%)**	347 (65.3)	308 (57.9)	249 (46.9)	<0.001	189 (64.9)	166 (56.8)	143 (48.8)	<0.001
**Age, years**	58 ± 13	59 ± 14	59 ± 13	0.215	56 ± 12	53 ± 12	52 ± 13	0.001
**DM, n (%)**	264 (49.7)	264 (49.6)	245 (46.1)	0.150	111 (38.1)	113 (38.7)	102 (34.8)	0.403
**Previous CVD history, n (%)**	117 (22.0)	85 (16.0)	84 (15.8)	0.001	35 (12.0)	44 (15.1)	43 (14.7)	0.679
**Modified CCI**	4.90 ± 2.17	5.03 ± 2.12	5.01 ± 2.10	0.442	4.37 ± 1.85	4.14 ± 1.86	4.11 ± 1.94	0.181
**SGA, n (%)**				<0.001				0.216
**Well-nourished**	447 (84.2)	439 (82.5)	375 (70.6)		185 (63.6)	228 (78.1)	212 (72.4)	
**Mild-moderate**	46 (8.7)	62 (11.7)	117 (22.0)		26 (8.9)	28 (9.6)	44 (15.0)	
**Severe malnourished**	0 (0)	0 (0)	0 (0)		1 (0.3)	0 (0)	1 (0.3)	
**Iron treatment, n (%)**				<0.001				0.132
**Oral iron treatment**	344 (64.8)	317 (59.6)	273 (51.4)		193 (66.3)	175 (59.9)	193 (65.9)	
**IV iron treatment**	43 (8.1)	56 (10.5)	88 (16.5)		4 (1.4)	3 (1.0)	7 (2.4)	
**No iron treatment**	142 (26.7)	165 (31.0)	167 (31.5)		93 (32.0)	107 (36.6)	9 (3.1)	
**Duration of dialysis, months**	33.1 (12.3–67.1)	33.5 (13.1–60.3)	33.8 (15.1–69.7)	0.222	36.7 (14.2–62.4)	32.0 (14.6–69.3)	25.7 (11.1–55.0)	0.026
**Systolic BP, mmHg**	140.1 ± 20.4	139.6 ± 19.5	143.1 ± 21.9	0.012	129.6 ± 16.9	134.0 ± 23.4	133.7 ± 22.9	0.031
**Diastolic BP, mmHg**	78.1 ± 12.1	76.6 ± 11.7	75.9 ± 14.0	0.016	79.1 ± 10.6	79.9 ± 13.5	79.3 ± 13.0	0.730
**Body weight, kg**	62.7 ± 11.1	59.5 ± 9.9	55.6 ± 9.6	<0.001	64.0 ± 11.8	63.8 ± 10.2	60.8 ± 10.8	0.178
ESA dose, *10 ^**^3**^ **U/week**	2 (0–4)	6 (6–8)	12 (10–15)	<0.001	0 (0–0)	4 (4–6)	8 (8–10)	<0.001
**ERI, U/kg/week/g/dl**	2.66 (0–5.12)	10.37 (8.31–12.61)	20.72 (17.46–26.81)	<0.001	0 (0–0)	7.03 (5.84–8.74)	13.74 (11.95–17.42)	<0.001
**Hemoglobin, g/dl**	11.4 ± 1.1	10.6 ± 1.0	10.0 ± 1.1	<0.001	11.7 ± 1.2	10.4 ± 1.2	9.8 ± 1.3	<0.001
**Iron, mcg/dl**	72.5 ± 33.1	70.3 ± 34.1	63.3 ± 32.2	<0.001	80.4 ± 36.3	70.6 ± 32.2	67.7 ± 30.9	<0.001
**TSAT, %**	32.1 ± 16.0	31.9 ± 15.7	30.6 ± 15.8	0.275	33.7 ± 15.6	30.3 ± 14.8	29.9 ± 14.9	0.020
**Ferritin, ng/ml**	233.4 ± 233.8	256.2 ± 268.6	383.9 ± 384.1	<0.001	294.2 ± 328.2	287.0 ± 316.2	279.7 ± 326.4	0.906
**Albumin, g/dl**	3.96 ± 0.38	3.92 ± 0.38	3.82 ± 0.40	<0.001	3.72 ± 0.45	3.69 ± 0.45	3.53 ± 0.48	<0.001
**Calcium, mg/dl**	8.90 ± 0.85	8.84 ± 0.88	8.69 ± 0.86	<0.001	8.74 ± 0.85	8.69 ± 0.82	8.59 ± 0.84	0.107
**Phosphorus, mg/dl**	6.65 ± 27.70	4.90 ± 1.46	4.81 ± 1.50	0.107	4.62 ± 1.41	5.01 ± 1.476	5.29 ± 1.57	<0.001
**Intact PTH, pg/ml**	201.2 ± 222.4	188.8 ± 223.5	202.5 ± 246.3	0.592	242.4 ± 200.4	328.1 ± 350.9	364.4 ± 395.6	0.001
**hsCRP, mg/dl**	1.37 ± 4.12	1.26 ± 4.85	2.32 ± 6.44	0.003	0.81 ± 3.48	0.47 ± 1.80	0.51 ± 1.46	0.273
**Total cholesterol, mg/dl**	153.6 ± 33.5	153.4 ± 37.3	151.3 ± 37.3	0.532	176.2 ± 36.1	177.2 ± 42.3	173.0 ± 42.0	0.467
**Ccr, ml/min**	1.11 ± 3.60	1.31 ± 4.80	1.27 ± 4.55	0.831	3.72 ± 7.65	1.62 ± 3.59	1.47 ± 3.38	<0.001
**Single-pool Kt/V**	1.45 ± 0.36	1.46 ± 0.35	1.51 ± 0.51	0.056				
**Weekly Kt/V**					1.56 ± 0.55	1.62 ± 0.45	1.74 ± 0.63	0.024

Data are expressed as means ± SD, medians (interquartile range) or numbers (percentages), as appropriate.

ERI, erythropoietin resistance index; BP, blood pressure; CVD, cardiovascular diseases; CCI, Charlson co-morbidity index; Ccr, creatinine clearance; ESA, erythropoiesis-stimulating agent; HD, hemodialysis; hsCRP, high sensitivity C-reactive protein; IV, intravenous; Kt/V: K, dialyzer clearance; t, time; V, volume of water a patient’s body contains; PD, peritoneal dialysis; PTH, parathyroid hormone; SGA, subject global assessment; TSAT, transferrin saturation.

### Relationship between ERI and All-Cause Mortality

The median follow-up period was 40 months (interquartile range, 23–54 months). During the follow-up period, 358 HD patients and 186 PD patients left the study. Among HD patients, the reasons for withdrawal included kidney transplantation (n = 98), transfer to a nonparticipating hospital (n = 158), refusal to participate further (n = 50), patient’s condition (n = 17) or others (n = 35). Among PD patients, the reasons for withdrawal included kidney transplantation (n = 77), transfer to a nonparticipating hospital (n = 53), refusal to participate further (n = 17), patient’s condition (n = 3) or others (n = 36). There were 159 deaths in HD patients and 135 deaths in PD patients during the follow-up period.

**Fig 2:**
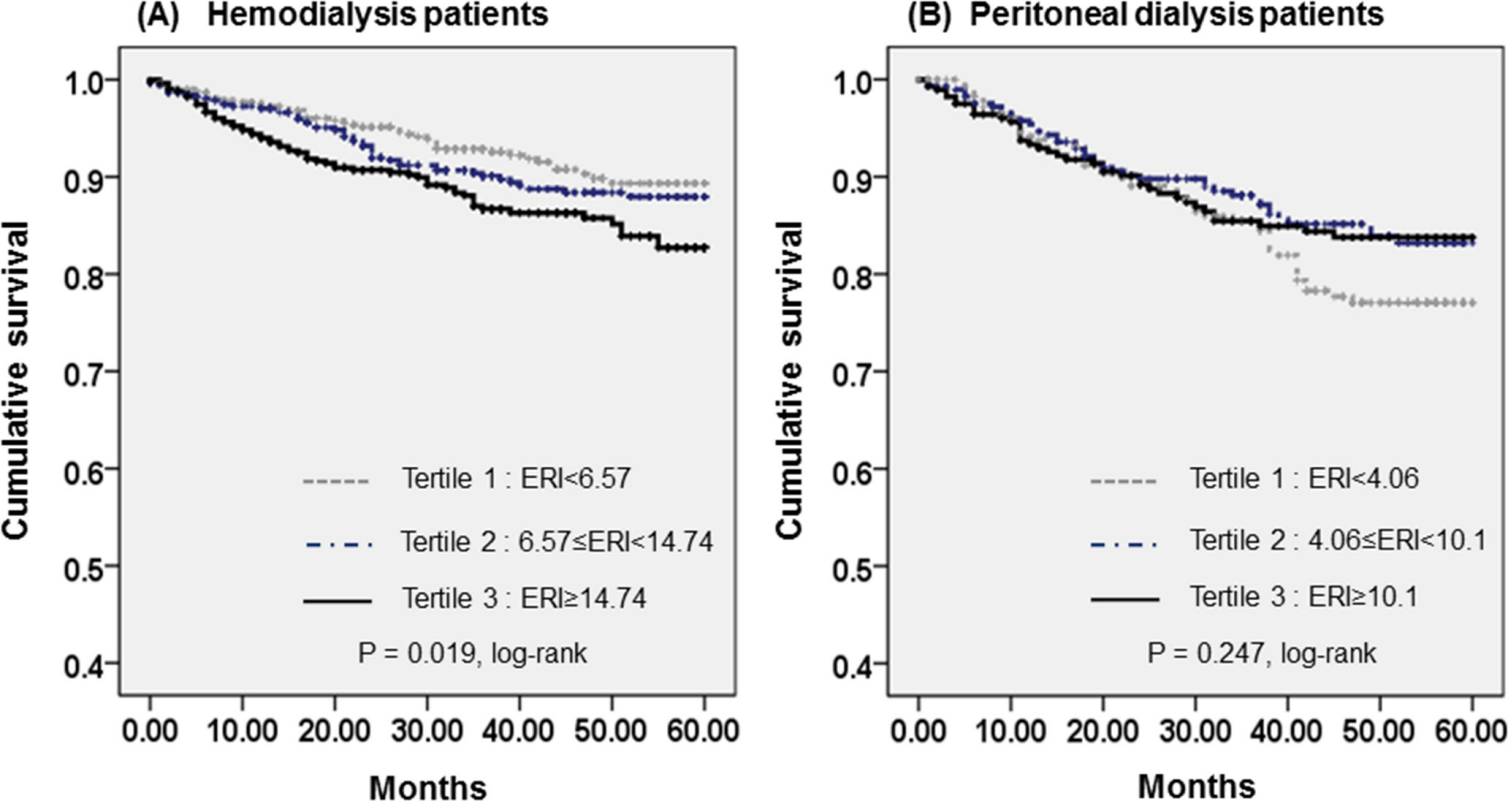
Kaplan-Meier plot of patient survival by tertiles of erythropoietin resistance index in (A) hemodialysis and (B) peritoneal dialysis patients.

The most common cause of death was cardiovascular disease (38.5% of all deaths) in HD patients and infectious disease (31.9% of all deaths) in PD patients. Table 3 shows the causes of death of the study population by tertiles of ERI. The absolute mortality rate was 3.28 deaths per 100 person-years in HD patients and 4.85 deaths per 100 person-years in PD patients. Fig 2 shows the Kaplan–Meier plot of patient survival by ERI tertile in HD and PD patients. In HD patients, all-cause mortality was significantly increased in the highest ERI tertile compared to those in lower ERI tertiles (*P* = 0.019, log-rank test). In PD patients, there was no significant difference in all-cause mortality among the three groups (*P* = 0.247, log-rank test).

**Table 3:** Causes of death of the study population by tertiles of ERI.

			HD			PD	
ERI		Tertile 1	Tertile 2	Tertile 3	Tertile 1	Tertile 2	Tertile 3
	Cardiovascular diseases including cerebrovascular diseases, n (%)	16 (39.0)	17 (35.4)	27 (40.3)	11 (20.4)	18 (43.9)	14 (35.0)
	Infectious diseases, n (%)	11 (26.8)	17 (35.4)	15 (22.4)	13 (24.1)	14 (34.1)	19 (47.5)
	Others, n (%)	14 (34.1)	14 (29.2)	25 (37.3)	30 (55.6)	9 (22.0)	7 (17.5)

ERI, erythropoietin resistance index; HD, hemodialysis; PD, peritoneal dialysis.

Univariate and multivariate Cox regression analyses for all-cause mortality in HD and PD patients are shown in Table 4. In HD patients, the crude HR for all-cause mortality in ERI tertile 3 was 1.70 (95% CI, 1.16 to 2.51, *P* = 0.007), when tertile 1 was used as the reference category. In multivariate Cox regression analysis, the adjusted HR for all-cause mortality in ERI tertile 3 was 1.96 (95% CI, 1.07 to 3.59, *P* = 0.029), even after adjusting for differences in demographics, laboratory data and co-morbid conditions. In PD patients, the crude HRs for mortality in ERI tertiles 2 and 3 were 0.73 (95% CI, 0.49 to 1.10, *P* = 0.135) and 0.76 (95% CI, 0.50 to 1.15, *P* = 0.194), respectively, using tertile 1 as the reference category. Even in multivariate Cox regression analysis, there were no significant differences in all-cause mortality among the tertiles: HR 1.13 (95% CI, 0.56 to 2.29, *P* = 0.741) for tertile 2, and HR 0.55 (95% CI, 0.21 to 1.43, *P* = 0.223) for tertile 3.

**Table 4:** Cox regression analysis of all-cause mortality.

HD					PD				
	Crude		Adjusted ^a^			Crude		Adjusted ^b^	
ERI	HR (95% CI)	P	HR (95% CI)	P	ERI	HR (95% CI)	P	HR (95% CI)	P
**Tertile 1**	1 (reference)		1 (reference)		**Tertile 1**	1 (reference)		1 (reference)	
**Tertile 2**	1.23 (0.82–1.85)	0.324	1.48 (0.76–2.84)	0.254	**Tertile 2**	0.73 (0.49–1.10)	0.135	1.13 (0.56–2.29)	0.741
**Tertile 3**	1.70 (1.16–2.51)	0.007	1.96 (1.07–3.59)	0.029	**Tertile 3**	0.76 (0.50–1.15)	0.194	0.55 (0.21–1.43)	0.223

^a^Adjusted for age, gender, diabetes mellitus, previous cardiovascular disease history, duration of dialysis, serum level of iron, ferritin, albumin, intact PTH, hsCRP, total cholesterol and single-pool Kt/V.

^b^Adjusted for age, gender, diabetes mellitus, previous cardiovascular disease history, duration of dialysis, serum level of iron, ferritin, albumin, intact PTH, hsCRP, total cholesterol and weekly Kt/V.

HD, hemodialysis; hsCRP, high sensitivity C-reactive protein; PD, peritoneal dialysis; ERI, erythropoietin resistance index; HR, hazard ratios; CI, confidence interval; P, P-value.

In addition to using the ERI, we also analyzed the relationship between all-cause mortality and ESA responsiveness based on a combination of ESA dosage and hemoglobin levels. Table 5 shows univariate and multivariate Cox regression analyses between categories of ESA responsiveness and all-cause mortality. In HD patients, patients with high-dose ESA and low Hb levels (ESA hypo-responsiveness) had a significantly higher risk of all-cause mortality, using the group with low-dose ESA and high Hb levels as a reference category: Crude HR 2.02 (95% CI, 1.32 to 3.10, *P* = 0.001) and adjusted HR 2.24 (95% CI, 1.16 to 4.31, *P* = 0.016). However, in PD patients, ESA hypo-responsiveness was not associated with all-cause mortality: Crude HR 0.79 (95% CI, 0.50 to 1.23, *P* = 0.294) and adjusted HR 1.75 (95% CI, 0.58 to 5.28, *P* = 0.319). There was no significant difference in all-cause mortality among the four groups (*P* = 0.129, log-rank test, data not shown).

**Table 5:** Hazard ratios for all-cause mortality by category of ESA responsiveness based on a combination of ESA dosage and hemoglobin level.

				Crude		Adjusted	
	ESA	Hb (g/dL)	N	HR (95% CI)	P	HR (95% CI)	P
**HD**	<6000^a^	<10	76	1.65 (0.77–3.53)	0.196	2.02 (0.56–6.99)	0.266
		≥10	582	1 (reference)		1 (reference)	
	≥6000^a^	<10	328	2.02 (1.32–3.10)	0.001	2.24 (1.16–4.31)	0.016
		≥10	608	1.73 (1.17–2.56)	0.006	1.83 (0.99–3.37)	0.053
**PD**	<4000^b^	<10	20	2.17 (0.93–5.08)	0.073	2.87 (0.84–9.87)	0.094
		≥10	293	1 (reference)		1 (reference)	
	≥4000^b^	<10	253	0.79 (0.50–1.23)	0.294	1.75 (0.58–5.28)	0.319
		≥10	310	0.95 (6.34–1.42)	0.798	1.11 (0.48–2.59)	0.802

Adjusted model included for age, gender, diabetes mellitus, previous cardiovascular disease history, duration of dialysis, serum level of iron, ferritin, albumin, intact PTH, hsCRP, total cholesterol and Kt/V.

^a^Median value of ESA dose in hemodialysis patients

^b^Median value of ESA dose in peritoneal dialysis patients.

ESA, erythropoiesis-stimulating agent; Hb, hemoglobin; N, number of patients; HD, hemodialysis; PD, peritoneal dialysis; HR, hazard ratios; CI, confidence interval; P, P-value.

## Discussion

In this multicenter prospective observational study performed in Korean ESRD population undergoing dialysis, we demonstrated that ESA responsiveness, calculated either by the ERI or by categorization based on combining ESA dose and Hb levels, was associated with all-cause mortality in HD patients, whereas it was not related to all-cause mortality in PD patients. Our findings suggest that the impact of the response to ESA treatment on all-cause mortality may be different between HD and PD patients.

The reasons why ESA responsiveness was not associated with all-cause mortality in PD patients in this study, while ESA hypo-responsiveness was associated with an increased risk of all-cause mortality in HD patients, are unclear. However, a number of explanations can be proposed.

First, as shown in Fig 1, the proportion of patients with high enough erythropoietin resistance to influence mortality in PD patients was so small that it may have resulted in the observed non-significant association between ERI and mortality in PD patients. A previous study reported that ESA requirements and ERI are lower in PD patients compared with HD patients [15–17]. Blood loss in HD sessions in HD patients may cause the increase in ESA requirements in HD patients [15]. In this study, similar to the results of the previous study, ERI was lower in PD patients compared with HD patients [24,25]. In HD patients, an ERI ≥ 14.74 U/kg/week/g/dL (tertile 3) was associated with all-cause mortality. The proportion of patients with an ERI ≥ 14.74 U/kg/week/g/dL in PD patients was only 18.9%. Therefore, the small proportion of patients with high enough erythropoietin resistance to influence clinical outcomes in PD patients may have contributed to the finding of a non-significant association between ERI and all-cause mortality.

Second, differences in the factors that condition the response to erythropoietin between HD and PD patients may have caused a different impact of ERI on mortality between HD and PD patients. ERI has been reported to be closely related with severe inflammation or malnutrition in HD populations [8,26]. The results of our study, similar to those reported in previous studies, demonstrated that ERI was positively correlated with an acute-phase response in the form of increased serum hsCRP levels and serum ferritin levels, and negatively correlated with SGA score and serum albumin levels in HD patients (Table 1). However, in PD patients, ERI was not significant correlated with SGA score, serum hsCRP and serum ferritin levels, although ERI was negatively correlated with serum albumin levels. These findings suggest that ERI more extensively reflects inflammatory and nutritional status, which are established risk factors for mortality in dialysis patients, in HD patients compared with PD patients. Therefore, it may be postulated that although ERI had prognostic value in HD patients, it had insufficient prognostic power to predict mortality in PD patients.

Third, differences in the administration route of ESA between HD and PD patients may have resulted in the different impact of ERI on mortality between HD and PD patients. As subcutaneously administered ESA such as Epoetin alfa or Darbepoietin alfa has a longer half-life than intravenously administered ESA, subcutaneous administration of ESA reduces the ESA requirements and can be more effective than intravenous administration [27,28]. As shown in Table 1, the most common ESAs used in this study are Epoetin alfa and Darbepoietin alfa. Subcutaneous administration of ESA is common in PD patients and consequently more prevalent in PD patients than HD patients, which reduces the ESA requirements in PD patients. It may be cautiously postulated that the lower ESA requirements and the lesser prevalence of resistance to ESA in PD patients may lessen the prognostic power of ERI on mortality.

For the association of ESA responsiveness with mortality in PD patients, our findings is not compatible with the results of previous studies reporting that low ESA responsiveness is associated with higher risk of mortality in PD patients [16,17]. This discrepancy may be due to differences of trends in anemia practice such as ESA use or the populations of the studies from previous studies [18,19]. In US study, usual ESA dose used in clinical practice was not associated with mortality and it was associated with higher risk of mortality when the ESA doses exceeded 15,000 U/week in PD patients [17]. In this Korean study, the percentage of patients with a weekly ESA dose > 15,000U/week was so small (1.1%), which may mitigate the impact of ESA responsiveness on mortality and resulted in disparity in ESA dose-mortality association between two the two studies.

In this study, the prevalence of previous CVD history at enrollment was significantly higher in the lowest ERI group than higher ERI group in HD (Table 2), which is inconsistent with the notion that higher ERI predict CVD in HD patients. Although the reason for this result is unclear, in consideration that old age is strong predictor of CVD in HD patients, we cautiously supposed that older age in tertile 1 may contribute higher prevalence of previous CVD history in baseline characteristics compared with higher tertles in HD patients (Table 2).

Our study has several limitations. First, the design of our study was not a randomized, controlled study. Second, in spite of the multicenter nature of the study, the cohort consisted of only Korean patients and all were Asian. Thus it is uncertain whether our results can be generalized to other ethnic groups with ESRD. Third, we could not analyze the effects of changes in ERI levels on all-cause mortality during the follow-up period because only baseline data were used.

In conclusion, our data show that ESA hypo-responsiveness was associated with increased risk of all-cause mortality in HD patients. However, ESA hypo-responsiveness was not related to mortality in PD patients. These findings suggest that the impact of ESA responsiveness on all-cause mortality is different between patients with HD and PD, and the interpretation of ESA responsiveness should be performed carefully in accord with the dialysis modality utilized.
